# Effects of Inhibition of Serine Palmitoyltransferase (SPT) and Sphingosine Kinase 1 (SphK1) on Palmitate Induced Insulin Resistance in L6 Myotubes

**DOI:** 10.1371/journal.pone.0085547

**Published:** 2013-12-23

**Authors:** Agnieszka Mikłosz, Bartłomiej Łukaszuk, Marcin Baranowski, Jan Górski, Adrian Chabowski

**Affiliations:** Department of Physiology, Medical University of Bialystok, Bialystok, Poland; Medical University of South Carolina, United States of America

## Abstract

**Background:**

The objective of this study was to examine the effects of short (2 h) and prolonged (18 h) inhibition of serine palmitoyltransferase (SPT) and sphingosine kinase 1 (SphK1) on palmitate (PA) induced insulin resistance in L6 myotubes.

**Methods:**

L6 myotubes were treated simultaneously with either PA and myriocin (SPT inhibitor) or PA and Ski II (SphK1inhibitor) for different time periods (2 h and 18 h). Insulin stimulated glucose uptake was measured using radioactive isotope. Expression of insulin signaling proteins was determined using Western blot analyses. Intracellular sphingolipids content [sphinganine (SFA), ceramide (CER), sphingosine (SFO), sphingosine-1-phosphate (S1P)] were estimated by HPLC.

**Results:**

Our results revealed that both short and prolonged time of inhibition of SPT by myriocin was sufficient to prevent ceramide accumulation and simultaneously reverse palmitate induced inhibition of insulin-stimulated glucose transport. In contrast, prolonged inhibition of SphK1 intensified the effect of PA on insulin-stimulated glucose uptake and attenuated further the activity of insulin signaling proteins (pGSK3β/GSK3β ratio) in L6 myotubes. These effects were related to the accumulation of sphingosine in palmitate treated myotubes.

**Conclusion:**

Myriocin is more effective in restoration of palmitate induced insulin resistance in L6 myocytes, despite of the time of SPT inhibition, comparing to SKII (a specific SphK1 inhibitor). Observed changes in insulin signaling proteins were related to the content of specific sphingolipids, namely to the reduction of ceramide. Interestingly, inactivation of SphK1 augmented the effect of PA induced insulin resistance in L6 myotubes, which was associated with further inhibition of insulin stimulated PKB and GSK3β phosphorylation, glucose uptake and the accumulation of sphingosine.

## Introduction

Sphingolipids belong to a group of lipid-derived molecules, comprising a sphingoid base as a backbone to which is attached a single fatty acid (FA) side-chain of varying length and degree of saturation [1,2]. Because these lipids are major constituents of cell membranes, for more than a century they were mainly considered to play a role in membrane integrity [3]. However, now it is clear that several sphingolipid metabolites including ceramide (CER), sphingosine (SFO) and sphingosine-1-phosphate (S1P) act as important signaling molecules and are implicated in a variety of cellular and physiological processes. Interestingly, despite the close structural homology of ceramide, sphingosine and S1P, the biological role of these lipids is different and in most cases even opposite [4]. Ceramide, the central molecule in sphingolipid structure and metabolism can accumulate in cells via two main routs: the hydrolysis of the membrane sphingomyelin, or its *de novo* synthesis from long chain fatty acids (LCFAs) [5-7]. The first and rate-limiting step of *de novo* synthesis is the condensation of a fatty acyl-CoA, usually palmitoyl-CoA, with serine, which is catalyzed by the enzyme serine palmitoyltransferase (SPT), to form 3-ketosphinganine [1,2,8]. The final two steps of this pathway involve the generation of dihydroceramide from sphinganine (SFA) by the action of dihydroceramide synthase and its subsequent conversation into ceramide by dihydroceramide desaturase [2,9,10]. In addition, the ceramide can be further modified into alternative forms, including glucosylceramide and ceramide 1-phosphate, or converted into other metabolites such as sphingosine 1-phosphate [2]. 

Recently, sphingolipids (SLs) have emerged as important mediators of insulin resistance (IR) [11]. Because, it is well established that the excessive delivery of palmitate results in substantial accumulation of ceramide, which interferes with insulin signaling pathways, causing IR [12-19]. Pharmacological or genetic inhibition of enzymes required for *de novo* ceramide synthesis (i.e., SPT, CerS) exerts a potent effect on cellular energetics and metabolism. Researches have demonstrated that acute inhibition of SPT using myriocin ameliorates the loss in insulin-stimulated protein kinase B (PKB/AKT) activation in cultured L6 or C2C12 myotubes [15,16,20-22]. These beneficial effects of myriocin are associated with reduced level of circulating ceramide and are reproduced when alternative inhibitors of *de novo* ceramide synthesis such as L-cycloserine (which also inhibits SPT) and fumonisin B1 (dihydroceramide synthase inhibitor) are used [21-23]. However, there is much less information regarding the long-term inhibition of key sphingolipid metabolic pathway enzymes and their participation in the development of IR. Moreover, there is also possibility that myriocin treatment may simultaneously reduce level of other sphingolipids derived from ceramide, thereby contributing to its beneficial effects. With this in mind, we sought to investigate the level of other sphingolipids, including SFA, SFO and S1P after short- (2 h) and long-term (18 h) myriocin treatments.

As well as targeting SPT directly, there is also some evidence to suggest that manipulating the activity of molecular targets or pathways that do not directly participate in the *de novo* synthesis of ceramide, may also result in the modulation of ceramide production [2,24]. Because breakdown of S1P is the only way for cellular lipids to exit the sphingolipid pathway, sphingosine kinase (SphK) is important in regulating the relative levels of CER/SFO and S1P. SphK exist in two distinct isoforms, SphK1 and SphK2, with SphK1 being dominant in skeletal muscle [24]. *In vivo* studies indicated that SphK1 gene delivery markedly reduced blood glucose level and greatly improved lipid profiles in KK/Ay diabetic mice [25]. Despite the clear proof that SphK1 activation prevents ceramide accumulation by promoting its metabolism into S1P [26], the role of SphK1 in regulating insulin action is still a matter of debate. Therefore, it is of interest to determine whether SphK1 is an important lipid kinase involved in regulation of glucose metabolism and whether inhibition of this enzyme affects sphingolipid profile in insulin resistan L6 myotubes. In an attempt to further understand and compare the role of SPT and SphK1 in fatty acid induced insulin resistance, the present study investigated the effects of palmitate on insulin action in L6 muscle cells in which SPT and SphK1 expression/activity was suppressed for the short (2 h) and long (18 h) term. 

## Materials and Methods

### Cell culture

All experiments were performed on L6 (rat-derived) myoblasts purchased from the American Type Culture Collection. L6 skeletal muscle cells were cultured according to methods described previously [27]. Briefly, L6 cells were grown and maintained in high-glucose DMEM (Dulbecco’s Modified Eagle’s Medium) supplemented with 10% FBS (Fetal Bovine Serum) and 1% antibiotic/antimycotic in a 5% CO_2_ humidified atmosphere at 37°C. Since the extent of myoblast fusion into myotubes declines with increasing passage, cells were used at low passages (between 2 and 5) for all experiments to maintain the differentiation potential of the cultures. Cells were cultivated to approximately 80% confluence in T75 culture flasks, trypsinized and seeded into 6 or 12-well culture plates for experiments. The cells were grown in the presence of 10% FBS until they reached about 80% confluency, at that time medium was replaced with DMEM containing 2% horse serum for the induction of differentiation into myotubes. Differentiation medium was changed every 48h. During this time, myoblasts fused to form elongated, multi-nucleated myotubes. Experiments were initiated when visual inspection confirmed that cells are differentiated (usually about 7 days, when greater than 90% of the cells were fused into myotubes).

### Cell treatment

The treatment dose of the inhibitors has been shown to inhibit palmitate-induced *de novo* ceramide accumulation in muscle and other cells [15,21,22,28]. Briefly, L6 muscle cells were pre-incubated with ceramide inhibitors i.e. 10 μmol/L of myriocin [a serine palmitoyltransferase inhibitor] and 10 μmol/L of SKI II [a sphingosine kinase 1 inhibitor] for 2 h before being incubated with 0.4 mM palmitate for 16 h. In some experiments, L6 myotubes were pre-treated for 2 h with above mentioned inhibitors and then subjected to incubation with 0.4 mM palmitate together with inhibitors for next 16 h. After this period cells were microscopically examined for abnormal cell growth or death (i.e. tryptan blue staining) due to specific treatment.

### Palmitate treatment

Palmitate was administered to cells by conjugating them with FFA-free BSA. Brieﬂy, palmitate was dissolved in ethanol and diluted 1:100 in DMEM (with 1% FBS) containing 2% BSA. L6 myotubes were incubated with the PA (ﬁnal concentration 0.4 mM) in 1% FBS–DMEM for 16 Fh. 

### 2-deoxyglucose uptake

The glucose uptake was conducted to assess responsiveness (sensitivity) of the cells to insulin. Cells were grown and differentiated in 12-well plates. Before determining glucose uptake, cell cultures were serum-starved in DMEM for 3h. Cells were washed three times at room temperature with Krebs-Ringer-Hepes buffer (140 mM NaCl, 20 mM Hepes, 5 mM KCl, 2,5 mM MgSO4, 1 mM CaCl2) and then were exposed to 100 nM insulin for 20 min. The uptake of glucose was assayed by incubating cells with 0,5 mM 2-deoxyglucose containing 1 µCi/ml [^3^H]-2-deoxyglucose for 10 minutes. Nonspecific tracer binding was determined by quantitating cell-associated radioactivity in presence of 10 µM cytochalasin B. Next the medium was removed and, cells were rinsed with ice-cold PBS (Phosphate Buffered Saline). Muscle cells were solubilized in 0,05 N NaOH. An aliquot of 50 µl cells solution was removed for protein determination via BCA method. The remaining fluid was placed in 5 ml vials and taken for liquid scintillation counting. 

### Protein extraction and Western Blot

Routine Western blotting procedures were used to detect protein content as described previously [29]. The cells were lysed in ice-cold RIPA (radioimmunoprecipitation assay) buffer (50 mM Tris-HCl, 150 M NaCl, 1 mM EDTA, 1% NP-40, 0,25% Na-deoxycholate, 1 mM phenylmethylsulfonyl fluoride, 1µg/ml aprotinin, 1 µg/ml leupeptin, 1 µg/ml pepstatin, 1 mM sodium orthovanadate, 1 mM sodium fluoride) and sonicated for 1 min at 4°C. Protein concentration was determined using BCA protein assay kit with bovine serum albumin as a standard. Samples were boiled at 95°C for 10 minutes in sample buffer containing 2-mercaptoethanol. Protein (60 µg) was subjected to SDS-PAGE and transferred to PVDF membranes, followed by blocking membranes in TTBS buffer (50 mM Tris-HCl, 130 mM NaCl and 0,05 % Tween-20) containing 5% nonfat dry milk for 90 min at room temperature. The membranes were then incubated overnight at 4°C with the corresponding antibodies at a dilution of 1:1000. Primary antibodies were purchased from Cell Signalling Technology [IRS1, phospho-IRS1 (Ser 302), Akt, phospho-Akt (Ser 473), GSK-3β, phospho-GSK-3β (Ser9)] and Novus Biologicals (β-tubulin). Thereafter the membranes were incubated with anti-rabbit IgG horseradish peroxidase-conjugated secondary antibody (1:3000; Santa Cruz Biotechnology, USA). Immunoreactive protein bands were visualized by using an enhanced chemiluminescence substrate (Thermo Scientific, USA) and quantified by densitometry (Biorad, USA). Equal protein concentrations were loaded in each lane as confirmed by Ponceau staining on the blot membrane. Protein expression was normalized to β-tubulin and reported as arbitrary units. Finally, the control was set to 100% and the experimental groups were expressed relative to the control.

### Lipid analysis

#### Concentration of sphingoid bases

Concentration of sphingosine, sphinganine, S1P was measured using the method previously described by Min et al. [30]. Briefly, internal standards (10 pmol of C17-sphingosine and 30 pmol of C17-S1P, Avanti Polar Lipids, USA) were added to the samples before sonication. The dried lipid residues were redisolved in ethanol and sphingoid bases were converted to their o-phthalaldehyde derivatives and analyzed using HPLC system (ProStar, Varian, USA) equipped with a fluorescence detector and C18 reversed-phase column (Varian, OmniSpher 5, 4.6 x 150 mm). The isocratic eluent composition of acetonitrile (Merck, Darmstadt, Germany) : water (9 : 1, v/v) and flow rate of 1 mL/min were used. The column temperature was maintained at 30°C. 

#### Concentration of ceramide

A small volume (50 µL) of the chloroform phase containing lipids extracted as described above was transferred to a fresh tube containing 40 pmol of N-palmitoyl-D-erythro-sphingosine (C17 base) as an internal standard. The samples were evaporated under a nitrogen stream, redissolved in 1,2 mL of 1M KOH in 90% methanol and heated at 90°C for 60 min to convert ceramide into sphingosine. This digestion procedure does not convert complex sphingolipids, such as sphingomyelin, galactosylceramide or glucosylceramide, into free sphingoid bases [31]. Samples were then partitioned by the addition of chloroform and water. The upper phase was discarded and the lower phase was evaporated under nitrogen. The content of free sphingosine liberated from ceramide was then analyzed using HPLC as described above. The calibration curve was prepared using N-palmitoylsphingosine (Avanti Polar Lipids) as a standard. The chloroform extract used for the analysis of ceramide contains small amounts of free sphingoid bases. Therefore, the concentration of ceramide was corrected for the level of free sphingosine determined in the same sample. 

### Statistical analysis

All data are expressed as mean ± SEM. Statistical difference between groups was tested with analysis of variance (ANOVA) and appropriate post hoc tests, or with a Student t-test. Statistical significance was set at p < 0.05.

## Results

### Effects of specific sphingolipid enzymes inhibitors (myriocin and SKI II) on glucose uptake in L6 myotubes

**Figure 1:**
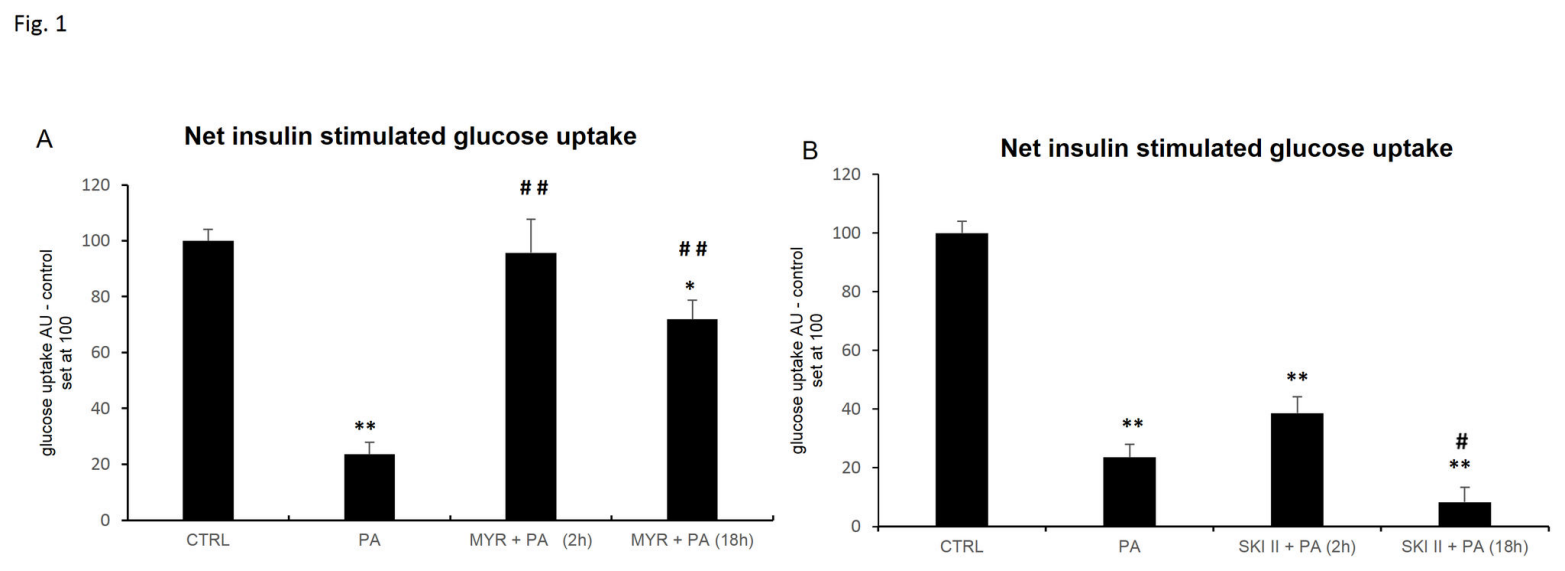
Net insulin-stimulated glucose uptake in L6 myotubes cultured with palmitic acid (PA) and (A) serine palmitoyltransferase inhibitor – myriocin (MYR) and (B) sphingosine kinase 1 inhibitor – SKI II. L6 myotubes were pre-incubated with 10 μmol/L of MYR or SKI II for 2 h [MYR/SKI II + PA (2 h)] and then incubated with PA (0,4 mM) for next 16 h. In some experiments, L6 myotubes were subjected to incubation with MYR or SKI II for 2 h and during the last 16 h incubation with ceramide inhibitors, cells were also incubated in the presence of palmitate as indicated [MYR/SKI II + PA (18 h)]. Cells were exposed to insulin (100 nM) for 20 min for assay to glucose uptake. Measurement of each treatment was taken as the average of six wells in the same experiments. Data are based on 3 independent determinations. Data are shown as mean ± SEM. *p < 0.05, **p < 0.01 significant difference: control (CTRL) vs. treatment. #p < 0.05, ##p < 0.01 significant difference: PA vs. treatment.

Based on the pilot studies, the maximal inhibitory effect of PA on insulin signaling in L6 myotubes was found at 0.4 mM (data not shown). As illustrated in Figure 1, PA reduced net insulin-stimulated glucose uptake (i.e. subtract glucose uptake under basal condition from that under insulin stimulation of the same treatment) by 77% (p < 0.05). For both 2 h and 18 h treatments with myriocin, we observed significant increases in the net insulin-stimulated glucose uptake (3-fold and 2-fold, respectively, Figure 1) as compared to that in PA-treated myotubes. In contrast, SKI II, a specific inhibitor of sphingosine kinase 1 reduced net insulin-stimulated glucose uptake by 65% (p < 0.05) after long-term (18 h) treatment as compared to PA. 

### Effects of specific sphingolipid enzymes inhibitors (myriocin and SKI II) on insulin signaling pathways (pIRS/IRS, pAKT/AKT, pGSK3β/GSK3β) and expression of SphK1

**Figure 2:**
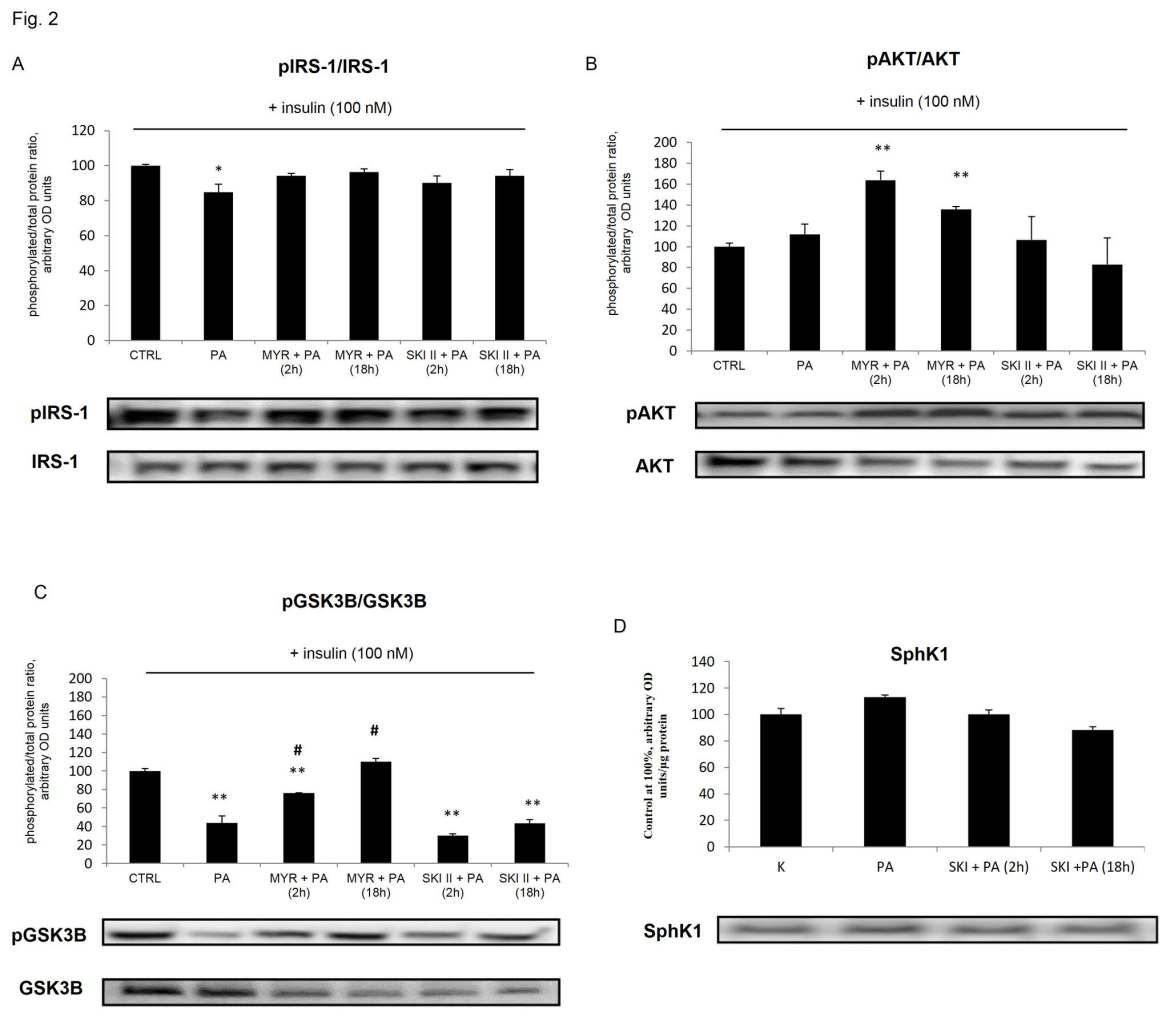
Protein expressions of (A) phosphorylated and total IRS1, (B) phosphorylated and total AKT, (C) phosphorylated and total GSK3B in L6 myotubes after either palmitate (PA), myriocin (MYR), SKI II or insulin exposure (100 nM, 10 min), (D) expression of SphK1 protein after palmitate and SKI II exposure. L6 myotubes were pre-incubated with 10 μmol/L of MYR or SKI II for 2 h [MYR/SKI II + PA (2 h)] and then incubated with PA (0.4 mM) for next 16 h. In some experiments, L6 myotubes were subjected to incubation with MYR or SKI II for 2 h and during the last 16 h incubation with ceramide inhibitors, cells were also incubated in the presence of palmitate as indicated [MYR/SKI II + PA (18 h)]. The samples were lysed in ice-cold RIPA buffer, and subsequent protein expression was measured as described in Methods. Measurement of each treatment was taken as the average of six wells in the same experiments. Data are based on 3 independent determinations. Data are shown as mean ± SEM. *p < 0.05, **p < 0.01 significant difference: control (CTRL) vs. treatment. #p < 0.05 significant difference: PA vs. treatment.

Correspondingly, we examined the consequences of the inhibition of SPT as well as SphK1 on insulin signaling. Incubation of muscle cells with 0.4 mM palmitate (for 16 h) induced a significant decrease in the insulin stimulated receptor substrate 1 (IRS1) serine phosphorylation (Figure 2A). By contrast, IRS1 serine phosphorylation, was not affected by either myriocin or SKI II (Figure 2A). However, we demonstrated that insulin stimulated AKT phosphorylation, which is downstream to IR signaling, was significantly improved in cells incubated with myriocin for 2 h and 18 h by 64% and 36%, respectively as compared to control (Figure 2B). Phosphorylation of (GSK3β), a physiological downstream AKT target, mirrored that of its upstream inactivating kinase. We observed that short and prolonged myriocin treatment noticeably increased the serine phosphorylation of GSK3β by 74% (p < 0.05) and 152% (p < 0.05) in L6 myocytes, compared with PA-treated cells (Figure 2C). In opposite, Figure 2C shows that palmitate induced the loss in phosphorylation of GSK3β by 56%, an effect that was further augmented following a 2 h incubation of cells with the SphK 1 inhibitor. We also analyzed the SphK1 protein content, which was not significantly changed after SKI II treatment (Figure 2D).

### Effects of specific sphingolipid enzymes inhibitors (myriocin and SKI II) on sphingolipids content in L6 myotubes

**Figure 3:**
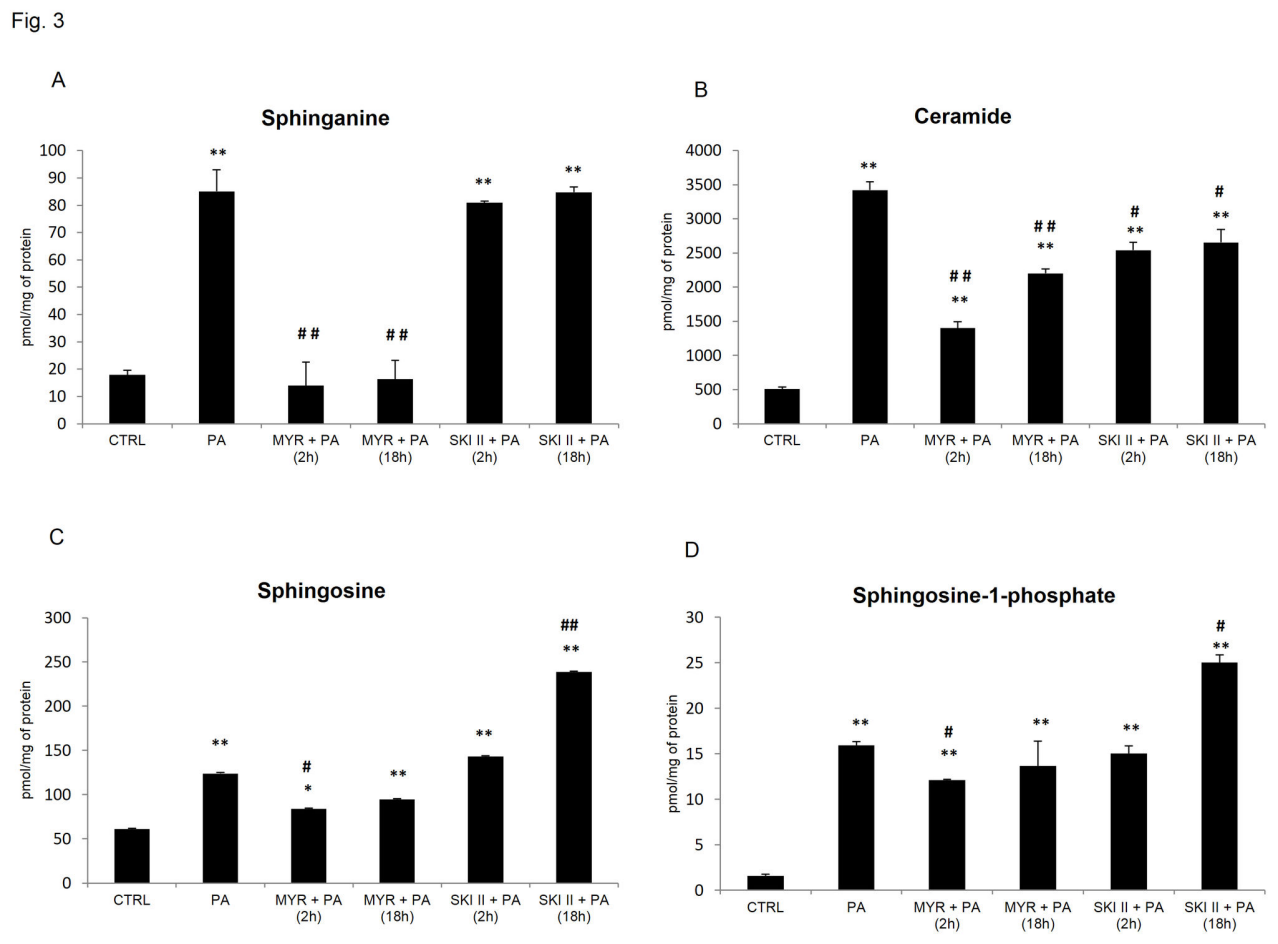
Effects of pharmacological (short and prolonged) inhibition of SPT and SphK1 on intracellular sphingolipids content in L6 myotubes. L6 myotubes were pre-incubated with 10 μmol/L of myriocin (MYR) or SKI II for 2 h [MYR/SKI II + PA (2 h)] and then incubated with PA (0.4 mM) for next 16 h. In some experiments, L6 myotubes were subjected to incubation with MYR or SKI II for 2 h and during the last 16 h incubation with ceramide inhibitors, cells were also incubated in the presence of palmitate as indicated [MYR/SKI II + PA (18 h)]. (**A**) Sphinganine (SFA), (**B**) Ceramide (CER), (**C**) Sphingosine (SFO), (**D**) Sphingosine-1-phosphate (S1P). The samples were harvested in ice-cold PBS buffer, and subsequent sphingolipid extraction was performed as described in Methods. Measurement of each treatment was taken as the average of six wells in the same experiments. Data are based on 3 independent determinations. Data are shown as mean ± SEM. *p < 0.05, **p < 0.01 significant difference: control (CTRL) vs. treatment. #p<0.05, ##p < 0.01 significant difference: PA vs. treatment.

Consequently, we assessed intracellular sphingolipids content in cells treated with myriocin and SKI II for 2 h or 18 h. Palmitate provision led to a substantial increase (~3,7-fold) in SFA level. However, in response to both short- (2 h) and long- (18 h) term treatments with the SphK 1 inhibitor, we did not observed any significant changes as compared to muscle cells incubated with palmitate alone (Figure 3A). In contrast, treatments of muscle cells with myriocin (for 2 h and 18 h) significantly lowered the SFA content in palmitate treated cells (Figure 3A). Further, CER content in PA-treated myotubes was 5,8-fold higher compared to control and this effect was reduced by 60% (p < 0.01) and 36% (p < 0.01), in the presence to both short and chronic treatments with myriocin (Figure 3B). Interestingly, SKI II, a specific inhibitor of sphingosine kinase 1, also decreased CER content in myotubes treated either for 2 h or 18 h with a combination of PA and SKI II, although to much lesser degree (26% and 22% p < 0.05) respectively (Figure 3B). Palmitate induced signiﬁcant elevation of the content of SFO, (100%, p < 0.01, Figure 3C) that was lowered by either short and prolonged myriocin treatments (32%, p < 0.05 and 24%, p > 0.05, Figure 3C). In contrast, 18 h incubation with SKI II inhibitor distinctly increased sphingosine content (92%, p < 0.01) as compared to that in PA-treated myotubes (Figure 3C). Palmitate induced an (9-fold, p < 0.01, Figure 3D) increase in S1P concentration and SPT inhibitor decreased the effect of PA (24%, p < 0.05 and 14%, p > 0.05 in response to both short and long treatments) (Figure 3D). On the contrary, SphK 1 inhibitor did not alter the effect of PA after short incubation, whereas chronic incubation further increased S1P content (by 57%, p < 0.05 as compared to that in PA-treated myotubes, Figure 3D).

## Discussion

In the present study, we compared for the first time effects of pharmacological (short and prolonged) inhibition of SPT and SphK 1 on palmitate induced insulin resistance in skeletal muscle cells *in vitro*. We found that myriocin improved insulin sensitivity both in the short- and long-term, whereas incubation with SKI II resulted in marked accumulation of sphingosine, a compound with the potential to promote IR within skeletal muscle. In addition, we showed that under potentially damaging conditions (such as PA treatment), SKI II significantly augmented the toxic effect of PA followed by inhibition of insulin stimulated PKB or GSK3β phosphorylation and glucose uptake. 

Based on a number of studies examining animal models of obesity and insulin resistance, a potential link between sphingolipids and metabolic disorders has been established [32]. The consequences of genetic and pharmacological inhibition of certain steps of sphingolipid metabolism indicate that sphingolipids play a major role in the pathogenesis of insulin resistance [33-35]. Ceramide is an attractive sphingolipid molecule that has been shown to accumulate in skeletal muscle of obese and insulin-resistant humans [17,36] and rodents [37,38]. Thus, inhibition of ceramide synthesis has emerged as a promising target for improving skeletal muscle insulin sensitivity. 

In this regard, we confirmed that prolonged exposure of L6 muscle cells to high palmitate concentration induced ceramide accumulation and profound inhibition of insulin-stimulated glucose transport [15,39]. Interestingly, short-term (2 h) inactivation of SPT by myriocin was sufficient to prevent ceramide accumulation even with prolonged (18 h) incubation with PA. It should be stressed that short-term inhibition of SPT totally reversed inhibitory effect of PA on insulin stimulated glucose uptake, which is in contrast to short-term inhibition of SphK 1, which only partially ameliorated the loss in insulin stimulated glucose uptake. Furthermore, prolonged inhibition of SphK1 intensified the insulin desensitizing effects of PA on glucose uptake in L6 myotubes. These findings are consistent with similar work reported previously using DMS (d-erythro-N,N-dimethylsphingosine), a potent SphK inhibitor, which completely abolished insulin effect in mouse C2C12 cells [25,40,41]. In fact, pharmacological or siRNA-mediated inhibition of SphK1 resulted in an appreciable decrease in basal and insulin-stimulated glucose uptake in C2C12 myoblasts [42]. Interestingly, it has been shown recently that SphK1 overexpression led to an enhancement in basal and insulin-stimulated glucose uptake in both 7721 and C2C12 muscle cell lines [25]. These results thereby indicate that SphK1 might be an important regulator of glucose metabolism. 

Saturated fatty acids such as palmitate elevated sphingolipid concentrations and subsequently induced insulin resistance via the inhibition of PKB or IRS1 phosphorylation [23,37]. Myriocin (a specific SPT inhibitor) significantly diminished the ceramide synthesis and reduced PKB activation, but it not interfere at the level of IRS-1 phosphorylation [15,18,35]. Moreover, myriocin also caused a significant increase in the pGSK3β/GSK3β ratio, indicating a trend towards increased muscle insulin sensitivity. Indeed, a number of independent studies have demonstrated that blocking ceramide accumulation using myriocin, cycloserine, or fumonisin B1 restores insulin-stimulated Akt and GSK3β phosphorylation, even in the presence of excess palmitate [20,37,43]. The present study demonstrated that short-term inhibition of SPT with myriocin was more effective in amelioration of palmitate-induced impairment in PKB phosphorylation than chronic inhibition of this enzyme. We postulated that, palmitoyl-CoA could not be used for *de novo* ceramide synthesis, palmitate might have been channeled into other pathways, such as DAG synthesis. In line with our hypothesis, Watson et al. [15] found that prolonged inhibition of SPT with myriocin elevated intramuscular DAG, and promoted a reduction in insulin-stimulated PKB phosphorylation. On the other hand, in our study, SKI II treatment clearly reduced pGSK3β/GSK3β ratio, exacerbating the negative impact of palmitate on insulin sensitivity. Therefore our findings suggest that alterations in manipulation of sphingolipid metabolism also at pathways other than *de novo* ceramide synthesis may represent an important factor, contributing to development of muscle insulin resistance.

Herein, we have also characterized the effect of saturated fatty acids (of which palmitate is the major component) and inhibition of SPT or SphK 1 on sphingolipid pools in skeletal muscle cells. The present study showed that myriocin significantly reduced the SFA level and totally reversed the insulin-desensitizing effects of PA. In contrast, SKI II (a potent SphK 1 inhibitor) induced insulin resistance in the muscle cells, which was related to an enhanced accumulation of sphingosine. In general, PA-induced accumulation of CER (as well as other sphingolipids) was to a large extent prevented by myriocin, indicating that the effect of PA on sphingolipid profiles results mostly from its use as a substrate for *de novo* sphingolipid synthesis [44-46]. Watson et al [15] have shown that ceramide generated *de novo* from palmitate is a major factor promoting insulin resistance in muscle cells and that short-term inhibition of SPT has beneficial effect on insulin signaling. Consistently with other reports [15,44], we also demonstrate that treatment with palmitate greatly increased (5.8-fold) the level of CER, which is diminished after myriocin treatment. Surprisingly, a recent study showed that, in contrast to short-term inhibition of SPT, its chronic suppression in L6 myotubes by pharmacological means or short-hairpin RNA-mediated silencing, fails to prevent the insulin-desensitizing effects of PA [2]. It was found that if SPT is chronically inhibited, the utilization of palmitate is diverted towards greater synthesis of DAG, which activates PKCs that in turn promote a reduction in IRS directed insulin signaling [15]. On the other hand, ceramide not only exerts multiple biological effects *per se*, but also serves as a precursor for the production of other bioactive sphingolipids, such as sphingosine and sphingosine-1-phosphate [47]. Degradation of ceramide is initiated by the action of ceramidase that produces sphingosine, which is then phosphorylated to S1P by SphK [24,48]. Interestingly, we demonstrated that treatment with myriocin lowered SFO concentration in myotubes as compared to that in PA-treated cells, whereas SKI II inhibitor has the opposite effect, increasing the level of sphingosine. Similarly, there is a study demonstrating that treatment with SKI II or DMS increases intracellular sphingosine, which inhibits PKC and thereby influences on glucose metabolism in skeletal muscle cells, hepatoma cells (SMMC – 7721) and MDA-MB cells [25,37,49,50]. However, Hu et al. [44] reported that SFO levels exhibited only little change upon PA treatment and no change in the presence of myriocin in C2C12 cells. It is interesting to note that S1P itself opposes the effects of ceramide on intracellular signaling, increasing the basal and insulin-induced glucose uptake *in vitro* [38,42]. The low level of S1P in cells is tightly regulated in a spatial manner by the balance between synthesis and degradation, which is the case for many other signaling molecules. Although, it can be speculated that, inhibition of SphK1 should significantly decrease S1P level, we did not detected such effect in L6 myotubes but rather an increase after 18 h inhibition of SphK1. Our results indicate that palmitate, which is considered as a crucial factor for the development of insulin resistance, can be metabolized by myocytes to S1P [51]. It has been previously reported, that SphK1 enzyme needs to translocate to the plasma membrane to catalyze the phosphorylation of its hydrophobic substrate sphingosine to S1P [7,52]. In turn, S1P may be quickly released to the extracellular environment, where it bound to the S1P2 receptor subtype [24,44,51,53]. Therefore, this bioactive sphingolipid may act in an autocrine mechanism via stimulation of the S1P2 receptor to diminish insulin signaling [51,52]. Additionally, it is know that SphK1 and SphK2 are two isoenzymes that use the same substrate and produce the same product, but have opposite functions in regulating sphingolipids metabolism and cell survival [24,52,54]. In our opinion, it is likely, that S1P formed under conditions of increased palmitate supply and concomitant inhibition by SKI II is produced mostly by SphK2 (this isoenzyme suppressed growth and enhanced apoptosis), but not SphK1. Moreover, S1P produced by SphK2 does not activate S1P receptors and thus can be accumulated in cell [54]. Finally, it is also likely that following addition of PA, both intracellular S1P and SFO content rapidly exceeded CER levels suggesting rapid dephosphorylation of S1P to SFO and subsequent reacylation to CER. In this regard, sphingosine can impair insulin signaling and might be responsible for the observed biological effects associated with increased ceramides. Although the results of our study cannot fully explain the exact mechanism by which the accumulation of CER impaired insulin pathway, both short- and long-terms inhibition of SphK1 intensified the palmitate induced insulin resistance in L6 myotubes. 

In conclusion, we showed that, SPT inhibition is more effective in restoration of palmitate induced insulin resistance than SphK1 in L6 myocytes. Importantly, SKI II significantly augmented the toxic effect of PA on inhibition of insulin stimulated PKB or GSK3β phosphorylation and glucose uptake, causing IR. Furthermore, we found that inhibition of SphK1 increased level of sphingosine, which can contribute to insulin resistance. 
